# Effects of Cisatracurium in Sevoflurane and Propofol Requirements in Dog-Undergoing-Mastectomy Surgery

**DOI:** 10.3390/ani12223134

**Published:** 2022-11-14

**Authors:** Claudia Interlandi, Simona Di Pietro, Giovanna L. Costa, Filippo Spadola, Nicola M. Iannelli, Daniele Macrì, Vincenzo Ferrantelli, Francesco Macrì

**Affiliations:** 1 Department of Veterinary Sciences, University of Messina, Polo Universitario Annunziata, Via Palatucci Annunziata, 98168 Messina, Italy; 2 Zooprophylactic Institute, Via Gino Marinuzzi 3, 90100 Palermo, Italy

**Keywords:** cisatracurium besylate, propofol, sevoflurane, dog, surgery

## Abstract

**Simple Summary:**

Gas anesthesia is widely used in clinical practice; it results in better management of the surgical patient by reducing the risks attached to anesthesia. Neuromuscular blockers, such as cisatracurium besylate, result in muscle relaxation of varying dose-dependent duration; however, they do not have analgesic and hypnotic efficacy, which is the reason of the combination with analgesic and anesthetic substances. The present study aimed to evaluate the possible reduction of anesthetic drugs used in bitches undergoing mastectomy surgery. The results obtained showed that the cisatracurium resulted in a good degree of myorelaxation without the manifestation of side effects and, more importantly, allowed the reduction of anesthetics used for induction (propofol) and maintenance (sevoflurane) of anesthesia. The possibility of reducing the doses of the drugs used maintaining also a good anesthesia enriches the scenario of different protocols that can be used in the canine patient.

**Abstract:**

The purpose of the present study was to test whether the addition of cisatracurium in combination with propofol and sevoflurane would result in a change in doses of used anesthetic drugs. Ten dogs (Group A) undergoing elective unilateral mastectomy surgery were included in the study. To induce and maintain anesthesia, subjects received propofol and sevoflurane at varying doses; analgesia was performed with remifentanil. After three months, the same subjects (Group B) underwent contralateral mastectomy and received the same anesthetic protocol with the addition of cisatracurium at a dosage of 0.2 mg/kg^−1^. The following parameters were monitored during anesthesia: heart rate, systolic blood pressure, end-tidal CO_2_, oxygen saturation, halogenate requirement, and rectal temperature at baseline (T^0^), induction (T^1^), 5 (T^5^), 10 (T^10^), 15 (T^15^), 20 (T^20^), 25 (T^25^), 30 (T^30^), and 35 (T^35^) time points. In Group A, halogenate requirement was reduced at all the time points other than T1 (*p* < 0.001); in Group B, the percentage of halogenate requirement was already reduced at T^1^ and remained constant during the experimental period, showing no significant intragroup differences. The dose requirements of sevoflurane and propofol varied significantly between the two groups, with significantly lower dosages in the Group B (the cisatracurium-treated group). Moreover, patients treated with cisatracurium showed a stable anesthetic plan. The nondepolarizing-muscle-relaxant cisatracurium besylate could be considered a useful adjunct to anesthetic protocols.

## 1. Introduction

The selection of anesthesia protocols during canine practice requires a careful assessment of the patient’s physical condition and an analysis of the nature or severity of ongoing disease processes. The application of a multimodal anesthetic protocol with the combination of different drugs is a balanced anesthetic technique, which can bring numerous advantages in patients [1,2].

In canine practice, neuromuscular-blocking agents (NMBs) have been used in different procedures: ophthalmological procedures (to prevent extraocular muscle contraction and produce a relaxed globe in a central position and reduce the risk of vitreous expansion), thoracotomy, laparotomy, ovariohysterectomy, castrations, diaphragmatic-hernia repair, portosystemic shunts, etc. [3,4,5]. Authors showed that NMB-induced neuromuscular blockade is potentiated and prolonged when halogenated anesthetics are used; in addition, inhalational anesthetics increase the neuromuscular blockade produced by nondepolarizing drugs in a dose-dependent manner [6,7,8]. Neuromuscular blocking agents cause myorelaxation but do not have hypnotic and analgesic effects, so they should always be administered with anesthetic and analgesic drugs [9,10,11].

Nondepolarizing drugs prevent acethylcoline (Ach) from binding to its receptors, preventing depolarization and muscle contraction. Cisatracurium has the advantage to undergo a spontaneous degradation in plasma by Hofmann’s hydrolysis, which is pH- and temperature-dependent, so it has a medium action. Its metabolism is independent of hepatic or renal function, which makes the drug particularly useful in individuals with impaired hepatic or renal function [7,12]. Cisatracurium does not trigger histamine release, so it does not produce a cardiovascular effect [2,13].

Cisatracurium has greater myorelaxant potency than atracurium, so it is used in lower doses. The reduction in dose results in lower plasma levels of laudanosine (active metabolite) and reduced side effects [8,12]. The purpose of this study was to evaluate the sevoflurane and propofol requirements and the vital parameters in dogs undergoing mastectomy, anesthetized with a multimodal regimen. The hypothesis was that the inclusion of the cisatracurium into anesthetic protocol could be the reason for the reduction of used anesthetic-drugs dosage, making the procedure more suitable in patients with a moderate anesthetic risk.

## 2. Materials and Methods

### 2.1. Animals

The study was conducted from 2011 to the year 2013. However, there was an update of the bibliography consulted. The study was approved by the Review Board for Animals Care of the University of Messina. All treatments, housing and animal care reported in this study were configured as a veterinary medical diagnostic clinical trial and were carried out in accordance with the EU Directive 2010/63/EU for animal experiments. Ten mixed-breed bitches with a mean age of 7.8 ± 1 (SD) years and mean weight of 18.4 ± 3.7 kg sterilized in the previous years were recruited for the study after written owner consent was obtained. The dogs were assigned American Society of Anesthesiologists (ASA) classification II based on clinical evaluation determined by physical examination, complete blood count, serum chemistry and radiological study.

Hematological and biochemical exams (blood urea, creatinine, glutamate pyruvate transferase, gamma glutamyl transferase, aspartate aminotransferase, and total protein) were within reference range for canine species. Mammary nodules were present in multiple mammary glands of both breast lines; the masses were similar in size and characteristics. X-ray and ultrasound examinations were performed before each surgery, showing no metastases in enrolled subjects. Inclusion criteria were sterilized medium-size adult female dogs affected by breast tumors sited on both breast lines that were going to undergo radical mastectomy of the entire breast line with lymphadenectomy of the axillar lymph node, corresponding to a physical status II according to the ASA classification. Subjects that showed mammary nodules in only one breast line or metastasis were not included in the study. Moreover, dogs affected by liver or renal insufficiency, obesity, peripheral-vessel or neuromuscular disease, or allergy to cisatracurium or propofol, were not included in the study.

### 2.2. Procedures

Food was withheld for at least 12 h prior, and water was withdrawn for 3 h prior to surgery. All patients underwent the anesthetic protocol following guidelines of WSAVA Global Pain Treatise. Dogs were divided in two groups: Group A (*n* = 10), including dogs undergoing a first monolateral radical mastectomy and anesthetized with a multimodal regimen including midazolam, propofol sevoflurane, and remifentanyl and Group B (*n* = 10), including the same dogs undergoing the second monolateral radical mastectomy, in which cisatracurium was added to the previous anesthetic protocol.

Cephalic veins were catheterized with a 20 or 18 gauge catheter (Farmacare SrL, Cannula, 20 gauge, 3.3 cm or 18 gauge, 4.5 cm; San Pietro in Casale (BO), Italy) for intraoperative fluid infusion and drug administration. Premedication was performed with midazolam 0.2 mg kg^−1^ intramuscularly (IM) (Ipnovel, 5 mg mL^−1^ Roche, Basel, Switzerland). Anesthesia was induced with propofol (Proposure 10 mg mL^−1^; Boehringer Ingelheim Animal Health Italia SpA, Noventana (PD), Italy) at the dosage of 4–6 mg kg^−1^ intra-venously (IV) to effect. In Group B, the administration of propofol was immediately followed by a single IV bolus injection of cisatracurium 0.2 mg kg^−1^ (Nimbex 2 mg mL^−1^ Glaxo, SmithKline SpA, Verona, Italy) [3,7,14].

The trachea was intubated using a cuffed endotracheal tube, and the dogs were connected to an anesthetic machine (Servo Ventilator 900C; Siemens-Elema, Grand Rapids, MI, USA); after intubation, the dog was placed in dorsal recumbency, and analgesic and saline solution were administered intravenously. Mechanical ventilation was performed with the following settings: respiratory rate of 16 breaths min^−1^ and a tidal volume of 10 mL kg^−1^, with an inspiratory duration of 25% and an inspiratory pause of 10%; a breathing circuit for dogs > 10 kg was used (LacK’s system, in parallel).

Anesthesia was maintained with sevoflurane (Sevoflo 100% Ecuphar Italia Srl, Milano, Italy) mixed in a carrier gas of 100% oxygen, delivered to the patients via an agent-specific out-of-circuit precision vaporizer. The concentration of sevoflurane requirement was adequate to provide a good surgical plan of anesthesia.

Intraoperative analgesia was provided by remifentanil hydrochloride (Ultiva 1 mg; GlaxoSmithKline SpA, Verona, Italy), mixed with saline solutions, IV injected via the right cephalic vein, using a syringe pump (Compact Syringe Driver, BRAUN, Germany and 0AJ 5803 Angel) with an initial constant rate infusion of 0.5 µg kg^−1^ min^−1^, at a concentration of 20 µg mL^−1^; the pump was calibrated according to the manufacturer’s specifications before the study.

Intravenous fluid administration of saline solution was started immediately following the induction at a rate of 5–10 mL kg^−1^ h^−1^ (Sodium chloride 9%, SALF SpA Cenate Sotto (BG), Italy).

The same surgical team performed all procedures.

The anesthetic depth was monitored evaluating physical signs, such as jaw tone, palpebral reflex, eyeball position, limb withdrawal reflex, and spontaneous movement.

A numerical cumulative pain scale (CPS), modified from previous studies [15,16,17,18,19,20], was performed by assigning a score on percentage variations of heart rate (HR, beats min^−1^) and systolic pressure (SAP mmHg) compared with basal values [20], according to the following procedure: (time point value − basal value/basal value) × 100 = % change. The score for both variables was then determined based on the following criteria: 

Score 0 ≤ 0%: basal value not changed

Score 1 ≥ 0% but ≤10%: basal value increased up to 10%

Score 2 ≥ 10% but ≤20%: basal value increased from 11% to 20%

Score 3 ≥ 20% but ≤30%: basal value increased from 21% to 30%

Score 4 ≥ 30%: basal value increased of more than 30%.

The same evaluator performed all procedures of pain assessment. The sum of the scores provides the total score; scores greater than 10 are indicative of inadequate analgesia. If the CPS scores were >10, an additional bolus of 0.5 to 1 µg kg^−1^ min of remifentanyl was given.

Postoperative analgesia was provided by buprenorphine 20 µg kg^−1^ (Bupaq Multidose 0.3 mg mL^−1^ Richter Pharma AG, Wels, Austria) IV administered at the end of surgery [21].

During the surgical procedure, the patient was connected to a multiparameter anesthesia monitor (AMI Italia s.r.l., Leonardo model, Milano, Italy), which continuously displayed heart rate (HR), plethysmographic oxygen saturation (SpO_2_), carbon dioxide at the end of expiration (EtCO_2_ mmHg), non-invasive systolic arterial pressure (SAP mmHg), and sevoflurane requirements (SR%).

Body temperature (T°) was monitored with a digital endorectal thermometer (GIMA Digital Thermometer, Milano, Italy). Patient monitoring included the assessment of above parameters at different time points: before drug administration (baseline values, T^0^), at the induction time (T^1^), and at 5 (T^5^), 10 (T^10^), 15 (T^15^), 20 (T^20^), 25 (T^25^), 30 (T^30^), and 35 (T^35^) min after drug injection. Time of extubation and achievement sternal recumbency were also monitored.

The evaluation of neuromuscular transmission was performed by detecting the TOF (train of four) using a machine (TOF-Watch^®^ SX, Organon, Italy) automatically set before each use at 50 mA and 1–0.1 Hz. The stimulating electrodes were applied at the medial part of the elbow (at the level of the ulnar nerve), whereas the recording of was obtained by applying the electrodes above the carpus. Stimuli were standardized by placing the electrodes in the same position on all dogs [7,19]. The peroneal nerve was stimulated and responses in the digital extensor muscles were observed. Electrodes with conducting gel (to optimize electrical contact) were applied at either site and used in conjunction with a peripheral nerve stimulator [12]. When the TOF value (T1:T4) was ≥0.7, we began weaning the patient off the ventilator.

If needed, blockages that showed a duration longer than reported for the canine species [5] could be antagonized with IV atropine 0.04 mg kg^−1^ (Atropine Solfato 1 mg mL^−1^ ATI, Italy) injected 1 min before 0.1 mg kg^−1^ neostigmine (Prostigmina^®^ 0.5 mg mL^−1^ MEDA Farma SpA, Milano, Italy). Potential side effects were appropriately treated.

Additional doses of cisatracurium 0.1 mg kg^−1^ were administered when the TOF (T1:T4) value was ≤0.5 and the surgery lasted longer.

### 2.3. Statistical Analysis

Statistical analysis was performed using SPSS 15.0 (IBM Company, Segrate, Italy). To assess the normal distribution of data, Shapiro-Wilk test was performed. Weight, age, and propofol dosage were expressed with mean ± standard deviation (SD) as normally distributed variables; anesthetic indices (HR, SAP, ETCO_2_, MAC, T°, extubation time, and station recovery) were expressed with median and range, as non-parametric data. The Wilcoxon test and *t*-test for paired data were used to compare the differences within the groups, along the time line; the Mann–Whitney U test and *t*-test for independent data were used to compare the differences between groups. A value of *p* < 0.05 was considered statistically significant.

## 3. Results

No side effect due to cisatracurium, such as prolonged action, bradycardia, hypotension, and bronchospasm were observed in all patients.

Sevoflurane requirement varied significantly in both groups, showing significantly lower rates in the cisatracurium-treated group (Group B) (*p* = 0.000). In Group A, the percentage of sevoflurane requirement was significantly reduced at all time points compared to baseline (*p* = 0.000) but still higher than Group B. The sevoflurane intra-Group B requirement was lower already at the time of induction (T^1^) and remained constant during all phases of anesthesia (SR = 1.24%) (Table 1).

The dose of propofol was significantly reduced in Group B, in which cisatracurium was administered, with a mean value of (2.49 ± 0.7) (SD) mg kg^−1^ compared with Group A, in which the muscle relaxant was not used, with a mean value of (6.95 ± 1) mg kg^−1^ and *p* = 0.000 (Table 2).

In Group A, HR values showed significant differences compared with T^0^, T^1^ (*p* = 0.005), T^5^ (*p* = 0.012), T^15^ (*p* = 0.005), T^20^ (*p* = 0.005), and T^25^ (*p* = 0.005). In Group B, the significant difference of HR values compared with T^0^ were T^5^ (*p* = 0.012), T^10^ (*p* = 0.032), T^15^ (*p* = 0.028), T^20^ (*p* = 0.028), T^25^ (*p* = 0.005); T^30^ (*p* = 0.018), and T^35^ (*p* = 0.018). The comparison of HR values between the two groups showed significant differences at T^10^, T^15^, T^20^, and T^25^ with *p* < 0.05.

Regarding SAP values, the Group A showed significant differences at all-time points from baseline, with a significance of *p* = 0.005. In Group B, significant differences were found at the following time points: T^1^ (*p* = 0.008), T^5^ (*p* = 0.012), T^20^ (*p* = 0.044), T^25^ (*p* = 0.012), T^30^ (*p* = 0.012), and T^35^ (*p* = 0.012) in comparison with T^0^. The comparison of SAP values between the two groups showed significant differences at all data points (*p* < 0.05), except at T^0^.

The carbon dioxide at the end of expiration (EtCO_2_) values showed no significant differences at all time points in Group A; in Group B, the comparison with T^1^ values showed significant differences at the following time points: T^5^ (*p* = 0.008), T^10^ (*p* = 0.007), T^15^ (*p* = 0.016), T^20^ (*p* = 0.024), T^25^ (*p* = 0.054), T^30^ (*p* = 0.021), and T^35^ (*p* = 0.005).

The SpO_2_ maintained optimal values in both protocols at all time points during anesthesia, with values of 98–99%.

The rectal temperature showed a decrease of about 1° in all dogs in both groups, with a mean value of 38.86 ± 0.3 at T^0^ and 37.92 ± 0.7 at T^35^ in Group A and 38.5 ± 0 at T^0^ and 37.4 ± 0.5 at T^35^ in Group B.

Physiological parameters were reported in Table 3.

Extubation time showed a significant difference between the two groups, with a median and range of 10 (7/14) min in Group A versus 2 (2/3) min in Group B (*p* = 0.000). The time of achievement of sternal recumbency also showed a significant difference between groups: 16.5 (13/20) min in Group A versus 5 (3/14) min in Group B, with *p* = 0.000 (Table 4).

## 4. Discussion

The results of this study showed that a single IV bolus of cisatracurium resulted in stable anesthesia and reduced the requirement of sevoflurane and dosage of propofol.

A different trend was observed within each group, as in Group A the reduction in halogenate requirement was noted only after induction, while dogs belonging to Group B showed an already reduced and constant halogenate requirement at the time of induction, indicative of an immediate adequate anesthetic plan that remained constant throughout the procedure (Table 1). Moreover, achievement of sternal recumbency was faster in the group treated with cisatracurium, showing a positive effect on recovery of all vital functions.

Cisatracurium is a nondepolarizing neuromuscular-blocking drug with a rapid onset time, medium half-life, and minimal cardiovascular effects [6]. Induction with cisatracurium is rapid, and this drug improved the intubation conditions. Moreover, the muscle relaxant allows a rapid adaptation to the anesthetic circuit with a good degree of hypnosis and good analgesia while maintaining lower doses of anesthetics, without hemodynamic repercussions [6,10,19,22].

In this study, a bolus dose of cisatracurium was given shortly after induction of anesthesia, before the introduction of the volatile agent, and a stable muscle relaxation was maintained. In accordance with other authors, the single bolus method has been preferred because it allows neuromuscular blockade to be constant throughout surgery [3,12,14].

Spontaneous degradation of NMb in plasma by Hofmann elimination is temperature-dependent: a variation > 2 °C in a patient’s body temperature during anesthesia could be causative of a variability in the drug half-life [3]. Therefore, particular attention must be given to the body temperature measurement in order to avoid side effects.

In this study, the dogs treated with NMb (Group B) had a temperature difference along the timeline of about 1 °C, thus reducing the possibility to have a variable duration of action of the neuromuscular blocker.

Some authors reported that an additional dose of cisatracurium (0.02 mg kg^−1^) provided a slight dose dependent cumulative effect [8]. In our study, it was not necessary to perform the increase of cisatracurium dosage at all time points, and there was no evidence of recurarization in the treated animals. Moreover, no dogs needed to reverse the muscle relaxant with atropine and neostigmine to achieve a rapid return of neuromuscular transmission [12].

A previous study reported that the onset time of NMbs could be affected by blood flow that is influenced by cardiac output and blood pressure. In fact, in animals with low cardiac output or low blood pressure, the onset of action may be delayed due to the vasodilation caused by some pre-anaesthetic medications [3]. In this study, the pre-anaesthetic midazolam, causing minimal cardiovascular changes at clinically relevant doses [23,24], allowed us to avoid this inconvenience. Although some studies reported behavioural alterations in dogs after the administration of midazolam alone, due to its excitatory effects, such as an increased motor activity associated with an intense sniffing [25,26], in the present clinical study, no subject showed similar side effects.

In the present study, remifentanil, a mu-opioid receptor agonist, was chosen for its analgesic- and anesthetic-sparing effect. It has a rapid onset and no tissue accumulation, even with prolonged administration. Other advantages of remifentanil are attributable to the lack of dependence on hepatic metabolism or renal excretion compared to other short-acting opioids currently used in combination with other anesthetics [19,20,21]. The use of remifentanil for pain control contributed to perform a good protocol due to its analgesic power and a very short duration of action. In addition, the pharmacokinetics of remifentanil has the great advantage of reducing recovery time [27,28,29].

Muscle-relaxant drugs have no known effect on the level of consciousness or pain threshold; therefore, they should always be combined with a good hypnotic and analgesic drug [5,10]. Conflicting data are present in literature about the role of propofol used in association with NMbs: some reports showed a prolonged effect of the muscle relaxant rocuronium after the propofol inoculation [11], while other studies did not report a similar effect [7,8]. In this study, the group treated with cisatracurium required a dosage of propofol lower than the untreated group, likely due to the facilitation of intubation induced by the muscle relaxant. We believe that the possibility to reduce the dosage of propofol, maintaining the inducing effects, is certainly preferable for a better management of anesthetized patients [30,31,32,33,34].

The reported difference in sevoflurane concentrations between the two groups is interesting (*p* = 0.000). The group not treated with cisatracurium needed higher doses of halogenate to achieve a good stage of anesthesia. Instead, the supplementation of the muscle relaxant probably contributed to the reduction of gaseous anesthetic doses throughout the anesthesia time [6,22]. Several authors have demonstrated a more pronounced potentiation of neuromuscular relaxant when administered with inhaled anesthetics such as sevoflurane or isoflurane, which increases skeletal muscle sensitivity to muscle relaxants [7,8,9,35]. During general anesthesia, one of the main goals is to maintain tissue perfusion and oxygenation, which depends on cardiac output and arterial oxygen content. The reduction of anesthetics’ doses improves these aspects, determining a good anesthetic plan [36]. Our results showed that the addition of cisatracurium in the anesthetic protocol appeared to be efficacious in reducing the propofol and sevoflurane concentration used in all dogs, which was ongoing to maintain an anesthetic- and analgesic-suitable plan for canine mastectomy surgery. This effect was clinically relevant, as showed by the improvement of vital parameter values during general anesthesia. No clinically evident changes in HR, SAP, T°, and EtCO_2_ measurements were identified during anesthesia. Comparing each time point with baseline (T0) values, significant different values of HR and SAP were observed in both groups, remaining within the physiological ranges of the canine species. During the entire anesthetic procedure, SAP showed significantly higher values, also closer to normal canine range, in the treated than untreated group. Moreover, carbon dioxide concentration at the end of expiration (ETCO_2_) showed values closer to hypocapnia (<30 mm/Hg) only at three time points in the treated group than untreated group, where these values were reached at almost all of the time points analyzed.

These findings support the hypothesis that a multimodal regimen that includes the cisatracurium can provide a good anesthetic and analgesic plan.

## 5. Conclusions

This study evaluated the changes of canine vital parameters as responses to the addition of cisatracurium during anesthesia and requirements of sevoflurane and propofol for canines undergoing mastectomy surgery.

Addition of a muscle relaxant to an anesthesiologic protocol (propofol, sevoflurane, and remifentanyl), in which the drugs used are all short-acting and easily adaptable to the patient’s demands could be an appropriate anesthesiologic protocol for mammary surgery in dogs. No adverse effects, other than muscle paralysis, were observed using cisatracurium. For these reasons, we recommend to include this NBM in anesthetic protocols for patients belonging to class ASA II subjected to a surgical procedure.

The limitation of the study is that these protocols were administered to only class ASA II patients. Further studies are needed to investigate these effects in healthy dogs and in dogs belonging to different ASA classes and to compare the parameters among different ASA statuses.

Finally, the opportunity to use low doses of anesthetic drugs represents a challenge not only for animal health but also for the environment. The influence of anaesthetic gases on global warming is being investigated, as some authors claim that volatile anaesthetics are halogenated compounds destructive to the ozone layer, so any protocol leading to a reduction of these drugs in addition to safeguarding animal welfare may also improve environmental pollution [37,38,39,40].

## Figures and Tables

**Table 1:** Concentration of sevoflurane requirements (SR %).

Variable	Time (min)									Significance between Groups
SR %	Groups	T1	T5	T10	T15	T20	T25	T30	T35	*p* = 0.000 ^†^
	A Group	5.6 (4.6/6)	3 * (2.7/3.5)	2.4 * (1.4/3.4)	3.1 * (2.2/4.2)	2.8 * (1.8/3.8)	3.2 * (2.2/4.2)	2.6 * (1.6/3.6)	2.6 * (1.6/3.6)	
	B Group	1.2 (1.1/1.3)	1.2 (1.1/1.3)	1.2 (1.1/1.3)	1.2 (1.1/1.3)	1.2 (1.1/1.3)	1.2 (1.1/1.3)	1.2 (1.1/1.3)	1.2 (1.1/1.3)	

Group A, after administration of midazolam 0.2 mg kg^−1^, propofol 6 mg kg^−1^, and remifentanil 0.5 µg kg^−1^ min^−1^; Group B, after administration of midazolam 0.2 mg kg^−1^, propofol 4 mg kg^−1^, and remifentanil 0.5 µg kg^−1^ min^−1^. * Significant difference compared to induction time (T^1^); ^†^ Significant difference between Group A and Group B; the data were expressed with median and range *p*-value.

**Table 2:** Requirement of propofol.

Variable	Group A	Group B	Significance
Propofolmg kg^−1^	6.95 ± 1 mg kg^−1^	2.49 ± 0.7 mg kg^−1^	*p* = 0.000 ^†^

^†^ Significant difference between Group A and Group B; the data were expressed with mean and SD *p* value.

**Table 3:** Physiological parameters.

Time (minutes)										
Variable	Groups	T0	T1	T5	T10	T15	T20	T25	T30	T35
HR (beats/min^−1^)	A Group	94 (86/97)	111 * (99/125)	100 * (92/108)	92 ^†^ (75/109)	74 *^†^ (65/83)	73 *^†^ (66/80)	70 *^†^ (64/76)	93 (70/115)	88 (67/101)
	B Group	94 (88/99)	109 (76/136)	103 * (92/118)	103 * (85/121)	84 * (70/98)	84 * (70/98)	78 * (68/88)	86 * (74/99)	84 * (69/99)
SAP (mmHg)	A Group	150 (110/158)	104 *^†^ (80/128)	99 *^†^ (87/111)	102 *^†^ (97/110)	100 *^†^ (94/110)	100 *^†^ (93/108)	107 *^†^ (96/124)	109 *^†^ (100/118)	108 *^†^ (98/118)
	B Group	135 (110/158)	145 * (126/164)	149 * (136/162)	129 (112/146)	127 (118/135)	127 * (112/132)	119 * (108/126)	121 * (114/127)	121 * (114/127)
ETCO_2_ (mmHg)	A Group		29 ^†^ (26/32)	28 (25/31)	28 (25/31)	29 (24/34)	28 (23/33)	29 (25/33)	30 (26/34)	30 (25/35)
	B Group		31 (29/33)	30 * (28/32)	29 * (28/32)	30 * (28/32)	30 * (29/32)	30 (28/32)	29 * (27/32)	28 * (27/29)
Body Temperature °C	A Group	38.6 (37.8/39.1)	38.1 * (38.1/38.2)	37.7 *^†^ (37.6/37.8)	37.7 ^†^ (37.6/37.8)	37.5 *^†^ (36.9/38.1)	37.2 *^†^ (36.4/38.1)	37.1 *^†^ (36.6/37.8)	37 * (36.3/37.0)	37.9 *^†^ (37.2/38.6)
	B Group	38.5 (38.4/38.6)	38.5 (38/39)	38.4 (38/38.8)	38 * (37.8/38.4)	38 * (37.8/38.4)	38.1 * (38/38.2)	37.8 * (37.2/38.2)	37.5 * (36.9/38.1)	37.4 * (36.9/37.9)

HR—heart rate; SAP—systolic arterial pressure; ETCO_2_—carbon dioxide at the end of expiration; T—body temperature, after administration of midazolam 0.2 mg kg^−1^, propofol 6 mg kg^−1^, and remifentanil 0.5 µg kg^−1^ min^−1^ (Group A); after administration of midazolam 0.2 mg kg^−1^, propofol 4 mg kg^−1^, and remifentanil 0.5 µg kg^−1^ min^−1^ (Group B); after induction time (T1); and at 5 (T^5^), 10 (T^10^), 15 (T^15^), 20 (T^20^), 30 (T^30^), and 35 (T^35^) min after drug injection. * Significant difference compared to baseline (T0); ^†^ Significant difference between Group A and Group B; the data were expressed with median and range *p*-value.

**Table 4:** Time extubation and achievement recumbency.

Variable	Group A	Group B	Significance
Time extubation (minutes)	10 (7/14)	2 (2/3)	*p* = 0.000 ^†^
Achievement of sternal recumbency (minutes)	16.5 (13/20)	5 (3/14)	*p* = 0.000 ^†^

^†^ Significant difference between Group A and Group B; the data were expressed with median and range *p*-value.

## Data Availability

The data presented in this study are available on request from the corresponding author.
